# Neutron scattering from myelin revisited: bilayer asymmetry and water-exchange kinetics

**DOI:** 10.1107/S1399004714023815

**Published:** 2014-11-22

**Authors:** Andrew R. Denninger, Bruno Demé, Viviana Cristiglio, Géraldine LeDuc, W. Bruce Feller, Daniel A. Kirschner

**Affiliations:** a Biology Department, Boston College, Chestnut Hill, MA 02467, USA; b Institut Laue–Langevin (ILL), CS 20156, F-38042 Grenoble CEDEX 9, France; c European Synchrotron Radiation Facility (ESRF), CS 40220, F-38043 Grenoble CEDEX 9, France; d NOVA Scientific Inc., Sturbridge, MA 01566, USA

**Keywords:** neutron scattering, myelin

## Abstract

Rapid nerve conduction in the central and peripheral nervous systems (CNS and PNS, respectively) of higher vertebrates is brought about by the ensheathment of axons with myelin, a lipid-rich, multilamellar assembly of membranes. The ability of myelin to electrically insulate depends on the regular stacking of these plasma membranes and on the presence of a number of specialized membrane-protein assemblies in the sheath, including the radial component, Schmidt–Lanterman incisures and the axo–glial junctions of the paranodal loops. The disruption of this fine-structure is the basis for many demyelinating neuropathies in the CNS and PNS. Understanding the processes that govern myelin biogenesis, maintenance and destabilization requires knowledge of myelin structure; however, the tight packing of internodal myelin and the complexity of its junctional specializations make myelin a challenging target for comprehensive structural analysis. This paper describes an examination of myelin from the CNS and PNS using neutron diffraction. This investigation revealed the dimensions of the bilayers and aqueous spaces of myelin, asymmetry between the cytoplasmic and extracellular leaflets of the membrane, and the distribution of water and exchangeable hydrogen in internodal multilamellar myelin. It also uncovered differences between CNS and PNS myelin in their water-exchange kinetics.

## Abstract 2

The structure of internodal myelin in the rodent central and peripheral nervous systems has been determined using neutron diffraction. The kinetics of water exchange in these tissues is also described.

## 1. Introduction   

The myelin sheath is a complex, multilamellar assembly of oligodendroglial or Schwann-cell plasma membranes that are spirally wrapped around axons, providing insulation and facilitating rapid signal transmission. Although relatively protein-poor compared with metabolically active membranes, myelin displays a surprising amount of heterogeneity, asymmetry and organization into structurally distinct functional domains, including the predominant internodal myelin, Schmidt–Lanterman incisures, the paranodal loops and – unique to the CNS – the radial component (Arroyo & Scherer, 2000 ▶; Scherer & Arroyo, 2002 ▶; Debruin & Harauz, 2007 ▶; Trapp & Kidd, 2004 ▶). These and related features have been the subject of intense investigation aimed at determining their normal composition, formation, organization and function. The wide variety of neurological disorders involving myelin provides additional challenges of determining how myelin fine structure is altered in diseases and the functional implications of these changes, and of assessing the extent and quality of structural and functional recovery in therapeutic paradigms.

Myelin structure has typically been investigated using light and electron microscopy (LM and EM, respectively) and X-ray diffraction (XRD). Although each method has its particular merits and limitations (Kirschner & Blaurock, 1992 ▶), XRD is especially useful because it can determine quantitative parameters about the structure of internodal myelin in a volume of unfixed tissue: *e.g.* the relative amount of myelin, its periodicity, the average membrane-bilayer profile and the packing of its membranes. The use of XRD to analyze fresh, unfixed tissue provides distinct advantages over LM and EM, both because of its higher spatial resolution and because the other techniques may require the use of potentially harsh chemical and/or physical treatments that alter myelin structure (Moretz *et al.*, 1969*a* ▶,*b* ▶; Kirschner & Hollingshead, 1980 ▶; Avila *et al.*, 2005 ▶).

With XRD, characteristic changes in the electron density of the membrane can be used to determine the localizations and widths of the most distinct structural features of intermodal myelin: the lipid polar headgroup layers, the hydrocarbon core of the bilayer and the aqueous spaces. While this analysis is comparatively straightforward, placing individual membrane components or specific chemical groups within the electron-density profile is much more difficult owing to the thermal disorder inherent in hydrated bilayers, the similarity in X-ray scattering power of three of the most abundant biological elements (O, C and N) and the fact that the profile represents the average of a heterogeneous membrane along the plane of the bilayer and through the stack of membranes (White & Wiener, 1995 ▶). Complete structural determination therefore requires additional biochemical or structural correlates. In diffraction experiments from artificial membrane systems, for example, this problem is addressed by localizing an increase in electron density in the bilayer owing to the specific labelling of membrane components with a heavy atom [*e.g.* bromine (Franks *et al.*, 1978 ▶; Hristova & White, 1998 ▶; Katsaras & Stinson, 1990 ▶; Lytz *et al.*, 1984 ▶; McIntosh & Holloway, 1987 ▶; MacNaughtan *et al.*, 1985 ▶; Wiener & White, 1991 ▶) or thallium (He *et al.*, 1993 ▶)]. Although widely used in the analysis of artificial membranes, this approach would be prohibitively difficult and potentially harmful to myelinated tissue.

Neutron diffraction (ND) can provide a complementary view of the membrane for a more complete understanding of bilayer structure. In contrast to X-rays, which are scattered by atoms in direct proportion to their number of electrons, neutrons are scattered by nuclei. The ability of an atom to scatter neutrons, as measured by its scattering length (*b*), depends on a complex relationship between its mass and nuclear energy levels. The result is that scattering lengths vary nonlinearly with atomic number and often vary significantly between isotopes of the same element. Owing to the large difference in neutron scattering lengths between deuterium (^2^H or D) and hydrogen (^1^H or H) (0.65 × 10^−12^ cm *versus* −0.38 × 10^−12^ cm, respectively; Bacon & Lonsdale, 1953 ▶) and to the fact that these atoms can be substituted for one another isomorphously, one can either highlight or suppress the scatter from particular features of a structure, potentially allowing the determination of molecular or atomic localization in natural membranes such as myelin.

The possibility of studying myelin using neutron diffraction was first demonstrated over 45 years ago (Parsons & Akers, 1969 ▶); however, there have only been a few follow-up studies directly addressing questions of myelin biology: Haywood and Worcester analyzed canine sciatic nerves to demonstrate the capability of a new neutron instrument (Haywood & Worcester, 1973 ▶); Kirschner and coworkers demonstrated hydrogen–deuterium contrast variation and H_2_O–D_2_O exchange kinetics in rabbit sciatic nerves (Kirschner *et al.*, 1976 ▶); Worcester and Ibel examined contrast variation in rabbit and frog sciatic nerves and presented low-resolution diffraction from rabbit optic nerves in D_2_O (as cited by Worcester, 1976 ▶); and Scott and coworkers attempted to determine the localization of deuterium-labelled cholesterol in rat sciatic nerves (Scott *et al.*, 1980 ▶). Although foundational, this research was severely limited by early neutron technology. Experiments required the use of large samples obtained from relatively large animals (*i.e.* dogs and humans) or bundles of nerves from smaller animals (*i.e.* rabbits and rats), multiple detector positions and exposure times as long as several days. For ND to be a useful technique for myelin research today, it must be rapid and compatible with single myelinated nerves from animals as small as mice, for which an ever-expanding library of neurologically relevant mutants exists and which therefore provide numerous therapeutic models. In the current paper, which presents a timely revisit of ND from myelin, we describe novel data from CNS myelin, improved data from PNS myelin, and water-exchange kinetics in both, and we discuss some immediate future possibilities for the analysis of myelin by neutron diffraction.

## 2. Materials and methods   

### 2.1. Specimens   

For neutron diffraction experiments, animals were housed at the Biomedical Facility at the European Synchrotron Radiation Facility, Grenoble, France, where all procedures were carried out. Optic nerves, spinal cords and sciatic nerves were obtained from mature C57BL/6J or C57BL/6 × 129S3/SvImJ mice (4–12 months of age; obtained from Charles River Laboratories, L’Arbresle, France, or provided by Dr A. Gow, Wayne State University Medical School) and Fischer (F344/IcoCrl) rats (four months of age; Charles River Laboratories) that had been sacrificed using isoflurane followed by decapitation. Spinal cords were routinely bisected sagittally before analysis. All samples were tied off at both ends with a silk suture and maintained in Tris-buffered saline (TBS; 5 m*M* Tris base, 154 m*M* NaCl, pH/pD 7.4) of varying D_2_O content (0–100%) until subsequent analysis. The knots on spinal cord segments were typically stabilized using cyanoacrylate adhesive because of the delicate nature of the tissue. For X-ray diffraction experiments, animals were housed and all procedures were carried out at the Boston College Animal Care Facility. Sciatic nerves and spinal cords were isolated from mature C57BL/6J mice (four months of age; obtained from Jackson Laboratory, Bar Harbor, Maine, USA) that had been sacrificed using CO_2_ asphyxiation and decapitation. Nerves were equilibrated against phosphate-buffered saline (PBS; 5 m*M* sodium phosphate, 154 m*M* NaCl, pH/pD 7.4) containing either 0 or 100% D_2_O. All animal procedures were conducted in accordance with protocols approved by the Institutional Animal Care and Use Committees of the respective institutions.

### 2.2. Neutron diffraction   

Neutron diffraction experiments were carried out on the D16 instrument at the Institut Laue–Langevin, Grenoble, France. To optimize sample illumination and angular resolution in the horizontal direction, we used two pairs of collimating slits. The resulting beam size at the sample was typically 1.5 mm (horizontal) × 15 mm (vertical). The neutron wavelength was 4.75 Å. Diffraction patterns were collected using the Millimeter Resolution Large Area Neutron Detector (MiLAND), a high-pressure ^3^He neutron detector with an area of 320 × 320 mm and a ‘pixel’ resolution of 1 × 1 mm. The sample-to-detector distances were either 870 or 900 mm. All neutron diffraction experiments were performed at ambient temperature (∼25°C) and pressure. For static measurements, samples were loaded into thin-walled quartz capillary tubes (Charles Supper Company, Natick, Massachusetts, USA) filled with TBS containing a known fraction of D_2_O and sealed with wax and enamel. Exposure times in static experiments ranged from 1 to 5 h. For H_2_O–D_2_O exchange experiments, the sample to be perfused was loaded into a Suprasil EPR tube (Wilmad-Labglass, Vineland, New Jersey, USA; inner diameter, 2 mm; outer diameter, 3 mm) that had been epoxied into a specially designed aluminium or Lucite yoke. Each end of the yoke had a stainless-steel pin for holding, *via* a tight-fitting O-ring, the suture attached to the end of the sample. The sample yoke was then sealed *via* O-rings into a Lucite perfusion yoke that was connected *via* Tygon tubing to a buffer reservoir and a peristaltic pump. The pump provided continuous replacement, at a flow rate of 0.5 ml s^−1^, of the fluid surrounding the sample.

Diffraction data for H_2_O–D_2_O exchange experiments were collected as a tandem series of increasingly long exposures: typically 12 × 5 s, 12 × 10 s, 12 × 15 s, 50 × 30 s and 30 × 1 min for a total exposure of ∼1 h. Longer exposures were routinely collected from each sample before and after exchange experiments to collect high signal-to-noise diffraction patterns and to ensure sample integrity; during exchange experiments, the intensity of the strong second-order reflection was sufficient to obtain time-resolved data. For most exchange experiments, we positioned the detector so that it could detect a range in *q* from −0.11 to 0.37 Å^−1^, which included the direct beam and each of the two second-order reflections near ±0.07 Å^−1^ (PNS) or ±0.08 Å^−1^ (CNS). This allowed improved counting statistics at low exposure times through the integration of the pair of unique second-order reflections. Our addition of a neutron-translucent, 250 µm thick, cadmium beamstop to attenuate the transmitted direct beam enabled us to collect sample transmissions during each data acquisition, so that the observed intensities at each time point in an exchange experiment could be properly corrected for transmission (which continuously changes during H_2_O–D_2_O exchange) and background.

### 2.3. Neutron data refinement   

All data refinement was performed using the ILL in-house software *LAMP* (http://www.ill.eu/instruments-support/computing-for-science/data-analysis). Firstly, the diffraction patterns were normalized to the incident neutron beam flux to account for variations in beam intensity and exposure time between samples. Secondly, they were normalized to a detector-calibration file containing the flat incoherent signal from water, which was used to correct the observed intensities for pixel efficiency and solid angle. Thirdly, they were corrected for the attenuation of the direct beam by the sample (transmission). Finally, background patterns from empty quartz capillaries or EPR tubes were collected and subtracted from each sample pattern. The corrected patterns were integrated along the fibre axis in either a window or azimuthally, depending on the sample type, to produce one-dimensional diffraction patterns. These resulting patterns were analyzed using either *LAMP* or *PeakFit* (Systat Software Inc.). The background was subtracted using local regression with Gaussian weighting. Reflections were fitted using asymmetric logistic peaks. The myelin period (*d*) was calculated from Bragg’s Law,
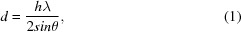
where *h* is the Bragg order for the observed reflection, λ is the neutron wavelength and θ is half of the scattering angle. The scattering vector *q* was calculated using
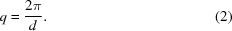
For samples analyzed at ≥43% D_2_O, the integrated intensity (*I_h_*) of the fourth-order reflection was reduced by *I*_2_^2^/*I*_0_ to remove the contribution of the doubly scattered strong second-order reflection (Kirschner *et al.*, 1976 ▶). In our experiments, the second-order intensity in 100% D_2_O was <0.1% that of the incident beam. Structure-factor amplitudes (|*F_h_*|) were calculated from corrected peak intensities by
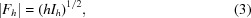
according to the beam geometry. For most samples, second-order structure factors were linearized *versus* %D_2_O and normalized to unity at 100% D_2_O to correct for small differences in sample size and orientation. The other structure factors were scaled based on the relative intensity of their second-order structure factors to the expected *F*_2_ at that %D_2_O. In the absence of a strong second-order reflection (*i.e.* at low %D_2_O), the fourth-order reflection was used instead. Consequently, any potential nonlinearity owing to extinction was removed from the dependence of *F_h_* on %D_2_O (Caspar & Phillips, 1976 ▶). Linear regression analysis was then performed between |*F_h_*| and %D_2_O. Phases were assigned to structure factors according to Kirschner *et al.* (1976 ▶). Average neutron scattering density profiles were calculated using *F_h_* derived from regression models with
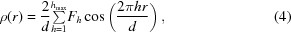
where *r* is the radial distance along the repeating unit (myelin membrane pair). Profiles were then scaled based on expected neutron scattering densities for myelin (Kirschner *et al.*, 1976 ▶; Kirschner, 1974 ▶). Uncertainty was modelled into the scattering density profiles according to Franks & Lieb (1979 ▶) using
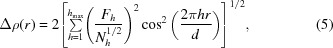
with the modification that Poisson counting statistics (*F_h_*/*N_h_* ^1/2^), where *N_h_* is the number of counts observed for a reflection of *h*th order, were used in place of Δ*F_h_* because of the lack of multiple measurements for some reflections. For unobserved reflections, counting statistics were extrapolated from measured values using linear regression.

### 2.4. X-ray diffraction   

X-ray diffraction experiments were performed as described previously (Avila *et al.*, 2005 ▶). Briefly, sciatic nerves and sagitally bisected segments of spinal cord were isolated from C57BL/6J mice and equilibrated against either D_2_O-saline or H_2_O-saline. They were then loaded into thin-walled quartz capillaries containing the same solution and sealed. X-ray diffraction was performed using nickel-filtered, single-mirror focused Cu *K*α radiation from a fine-line source on a 3.0 kW Rigaku X-ray generator operated at 40 kV and 10 mA. Exposure times were 30 min. Diffraction patterns were collected using a linear, position-sensitive detector (Molecular Metrology, Northampton, Massachusetts, USA) and were analyzed using *PeakFit*. All X-ray diffraction experiments were performed at ambient temperature (∼22°C) and pressure. The myelin period was calculated from the positions of the intensity maxima in the diffraction patterns. The relative amount of myelin [M/(M + B)] was calculated by comparing the integrated intensity of all maxima with the total scatter, including the background and excluding small-angle scatter around the beamstop and wide-angle scatter (Avila *et al.*, 2005 ▶). Relative amounts of myelin were then scaled to correct for differences in diffraction intensity originating from lattices with different periods (Kirschner & Caspar, 1975 ▶).

## 3. Results and analysis   

### 3.1. Neutron diffraction from rat and mouse PNS myelin   

**Figure 1:**
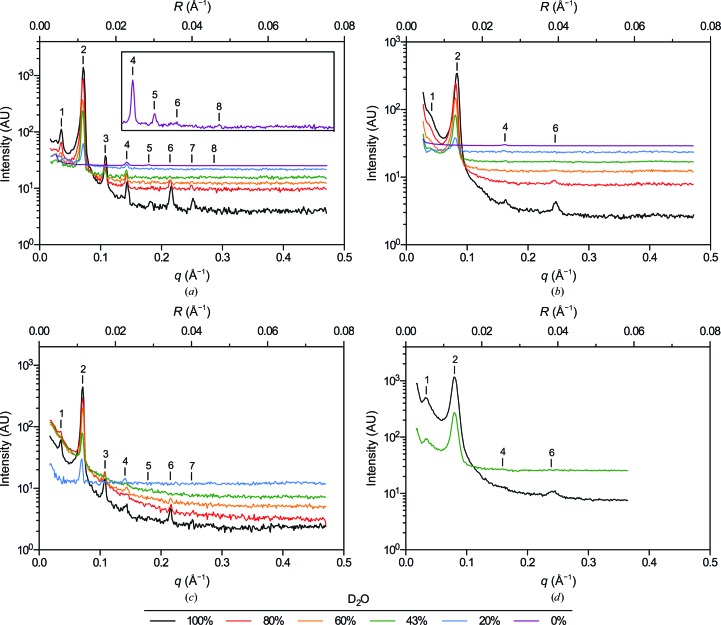
Neutron diffraction patterns from (*a*) rat sciatic nerves, (*b*) rat optic nerves, (*c*) mouse sciatic nerves and (*d*) mouse spinal cords equilibrated against 0–100% D_2_O-saline. Scattering intensity is plotted against both the scattering vector *q* (2π/*d*; Å^−1^) and the reciprocal coordinate *R* (1/*d*; Å^−1^). Bragg orders are indicated with numerals above the reflections. The inset in (*a*) is expanded along the *y* axis to more clearly show the additional Bragg orders recorded in 100% H_2_O.

**Figure 2:**
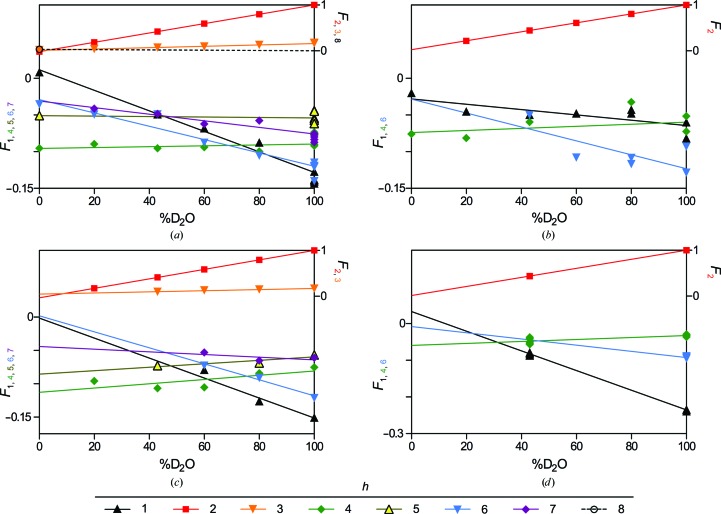
Structure-factor amplitudes with assigned phases *versus* %D_2_O for (*a*) rat sciatic nerves, (*b*) rat optic nerves, (*c*) mouse sciatic nerves and (*d*) mouse spinal cords. Symbols represent experimental replicates. Lines represent the linear dependence of *F_h_* on %D_2_O. In the absence of measurements of *F*_8_ in rat sciatic nerve at multiple concentrations of D_2_O, the linear regression of *F*_8_ *versus* %D_2_O was forced through 0 at 100% D_2_O. Similarly, *F*_6_ for mouse spinal cord was modelled using the single observed reflection at 100% D_2_O-saline and the slope of *F*_6_ *versus* %D_2_O from rat optic nerve. The *y*-axis labels are colour-coded to correspond to the respective data sets.

To benchmark current ND capabilities for myelin structure analysis, and because no data have been presented since the original research from the 1970s, we first examined rat sciatic nerves and compared these results with the early data collected from rabbit peripheral nerves (Kirschner *et al.*, 1976 ▶). Although a single rat sciatic nerve (<1 mm in diameter; >15 mm in length) is significantly smaller than the bundles of rabbit sciatic nerves previously analyzed (three each of >1 mm in diameter), it produced a series of strong Bragg reflections (Fig. 1 ▶ *a*) corresponding to an average myelin period of 175.5 ± 1.5 Å (*n* = 11), which is typical for rodent PNS myelin in X-ray diffraction experiments (Kirschner & Blaurock, 1992 ▶). Varying the D_2_O content of the sample resulted in significant changes in the neutron diffraction patterns. With decreasing %D_2_O the intensities of the observed reflections generally decreased, although not uniformly among Bragg orders, and the incoherent background level rose as the amounts of hydrogen (^1^H) increased in the sample. Data collected at low %D_2_O generally required higher counting times to obtain reasonable counting statistics. We observed seven reflections from rat sciatic nerve in 100% H_2_O-saline (Bragg orders 1–6 and 8; Fig. 1 ▶ *a*, inset). Otherwise, Bragg orders 1–7 were typically observed, including the occasional presence of the fifth-order reflection which had previously been unresolved even at high %D_2_O (Kirschner *et al.*, 1976 ▶). The relative intensities of the reflections were consistent with previous findings, particularly the dominant second-order reflection in high %D_2_O, which is accounted for by the two distinct water layers (*i.e.* the cytoplasmic and extracellular compartments) within the multilamellar array. As expected, the calculated structure factors varied linearly with the concentration of D_2_O in the sample (Franks & Lieb, 1979 ▶; Fig. 2 ▶ *a*, Table 1 ▶).

**Figure 3:**
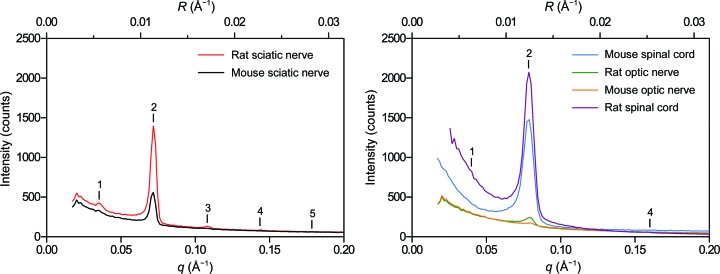
Comparison of neutron diffraction from rat and mouse (left) peripheral and (right) central nervous system tissue. Neutron diffraction patterns have only been corrected for detector efficiency, solid angle and exposure time to demonstrate the relative intensities of the second-order Bragg reflection from the respective samples. Scattering intensity is plotted against both the scattering vector *q* (Å^−1^) and the reciprocal coordinate *R* (Å^−1^). Bragg orders are indicated with numerals above the reflections.

The limit of recording ND from PNS myelin was explored by examining even smaller samples: nerves dissected from mice. Mouse sciatic nerves (<0.5 mm in diameter; >15 mm in length) equilibrated against buffered saline containing varying amounts of D_2_O yielded reflections corresponding to an average myelin period of 175.3 ± 2.2 Å (*n* = 5; Fig. 1 ▶ *c*). Similar to rat PNS myelin, the second-order reflection dominated at high %D_2_O. Lesser contributions from Bragg orders 1 and 3–7 were also observed. Diffraction from mouse sciatic nerves was significantly weaker than that from rat sciatic nerves; *I*_2_ for mouse nerves was about one-third of that from rat nerves (Fig. 3 ▶). Nonetheless, analyzable data were collected even at low %D_2_O, and the same linear relationship between *F_h_* and %D_2_O was observed (Fig. 2 ▶ *c*, Table 1 ▶).

### 3.2. Neutron diffraction from rat and mouse CNS myelin   

**Figure 4:**
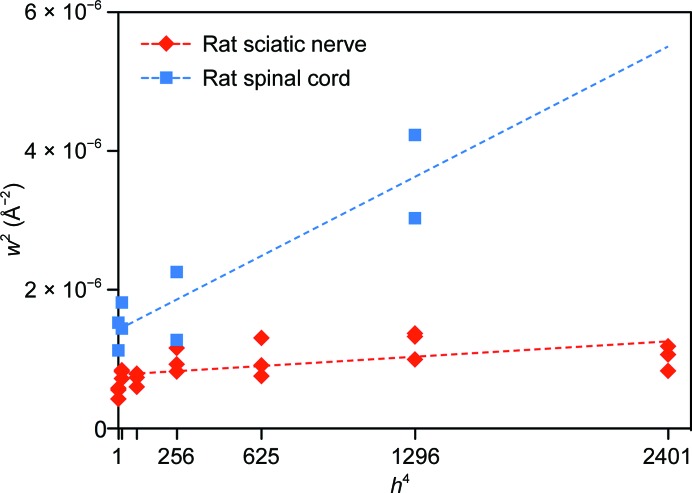
Comparison between rat spinal cord (CNS) and sciatic nerve (PNS) myelin packing disorder and the crystallinity of myelin. The square of the full-width at half-maximum for each reflection (*w* ^2^) is plotted against the fourth power of the Bragg order (*h* ^4^). Dashed lines behind the data represent linear least-squares fits for spinal cord (blue) and sciatic nerve (red). The slope of each line is directly related to the amount of membrane-packing disorder in the tissue, while the intercept is inversely proportional to the crystallinity (the coherence length or the average number of layers of myelin; Inouye *et al.*, 1989 ▶). The coherence lengths for spinal cord and sciatic nerve were approximately eight and 12 repeats (membrane pairs), respectively.

Previous work suggested that CNS myelin had only a limited capacity to provide meaningful neutron scattering data (Worcester, 1976 ▶). To determine the extent to which ND could be used to analyze CNS myelin, we examined rat optic nerve and spinal cord (2 mm in diameter; >15 mm in length), followed by mouse optic nerve and spinal cord. Because of their small size (∼0.5 mm in diameter; <10 mm in length), we expected rat optic nerves to diffract much more weakly than sciatic nerves. It also has been previously demonstrated using XRD (Avila *et al.*, 2005 ▶ and subsequently by our own ND experiments) that diffraction from CNS myelin is generally of lower quality than that from PNS myelin because of the smaller coherent domain size (fewer layers of myelin) and increased disorder in membrane packing in CNS myelin (Fig. 4 ▶; Inouye *et al.*, 1989 ▶). We found, however, that diffraction from rat optic nerves was of sufficient intensity and quality for meaningful analysis, even at low %D_2_O (Fig. 1 ▶ *b*). Depending on the D_2_O content of the nerves, Bragg orders 1, 2, 4 and 6 were visible and indexed to a myelin period of 155.0 ± 1.1 Å (*n* = 8), typical of rodent CNS myelin (Kirschner & Blaurock, 1992 ▶). The second-order Bragg reflection dominated the patterns at high %D_2_O (with ∼15% of the intensity of the second-order reflection from rat sciatic nerve), indicating the presence – similar to PNS myelin – of two distinct water layers within the multilamellar array. Very weak first-, fourth- and sixth-order reflections were also detected. In contrast to the diffraction from PNS myelin, the absence of the higher odd-order reflections (*h* = 3, 5 and 7) from CNS myelin demonstrates that the aqueous spaces at the extracellular and cytoplasmic appositions have similar widths. As for PNS myelin, the calculated structure factors varied linearly with %D_2_O (Fig. 2 ▶ *b*, Table 1 ▶).

Although routinely used for X-ray diffraction (see, for example, Avila *et al.*, 2005 ▶), mouse optic nerves (<0.5 mm in diameter; ∼5 mm in length) diffracted neutrons very poorly even in 100% D_2_O-saline, yielding a faint first-order reflection and a second-order reflection that was <5% of the intensity of that of rat sciatic nerve (Fig. 3 ▶). At 20% D_2_O-saline (not shown), measurable diffraction was not detected. By contrast, diffraction from segments of bisected spinal cord (∼1.5 mm in diameter; >15 mm in length; Fig. 1 ▶ *d*) was strong, with the intensity of the dominant second-order reflection being approximately twofold more intense than that of rat sciatic nerve under identical conditions (Fig. 3 ▶). Weak but distinct Bragg orders 1, 4 and 6 were also observed. The average myelin period for mouse spinal cord was 156.3 ± 0.6 Å (*n* = 8). Again, the absence of higher odd-order reflections demonstrated the similar widths of the cytoplasmic and extracellular appositions, which has been demonstrated by XRD. The linear relationship between *F_h_* and %D_2_O for mouse spinal cord is shown in Fig. 2 ▶(*d*) and Table 1 ▶.

### 3.3. Structure of CNS and PNS myelin   

**Figure 5:**
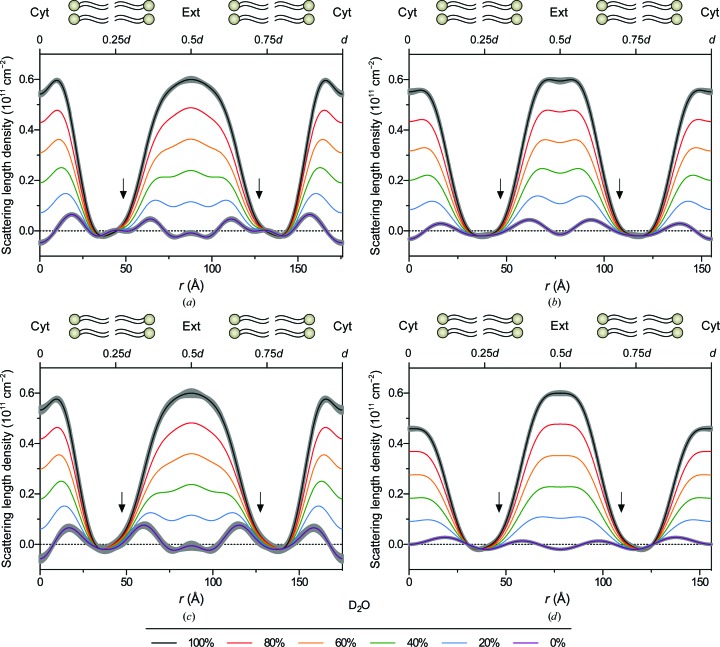
Neutron scattering length density profiles from (*a*) rat sciatic nerves, (*b*) rat optic nerves, (*c*) mouse sciatic nerves and (*d*) mouse spinal cords in 0–100% D_2_O-saline. Scattering length density is plotted against radial distance *r*, with the centre of the cytoplasmic apposition at *r* = 0. For clarity in the bilayer regions, uncertainty (grey borders) was included only for the profiles calculated for myelin in 0 and 100% D_2_O-saline. The arrow indicates the higher level of neutron scattering density in the extracellular half of the bilayer, which is proposed to relate to an asymmetric distribution of cholesterol. For each panel, the upper *x* axis indicates the positions of 0.25*d*, 0.5*d*, 0.75*d* and *d*.

Neutron scattering density profiles were calculated for myelin from rat sciatic and optic nerves and mouse sciatic nerves and spinal cords (Fig. 5 ▶). For both PNS (Figs. 5 ▶ *a* and 5* ▶c*) and CNS myelin (Figs. 5 ▶ *b* and 5 ▶ *d*) at high %D_2_O, two regions of high neutron scattering density characterized the profiles, centred at *r* = 0 and *r* = 0.5*d* and corresponding to the two distinct aqueous spaces within myelin: the cytoplasmic and extracellular compartments, respectively. As D_2_O was replaced with H_2_O, these spaces displayed dramatic changes in scattering density owing to the high proportion of exchangeable hydrogen in water and in other constituents. Between this pair of aqueous compartments were regions of relative constancy that were largely unaffected by alterations in H/D content. These stable regions, near *r* = 0.25*d* and *r* = 0.75*d*, correspond to the hydrocarbon layers in the membrane, which exclude water and are rich in nonexchangeable hydrogen. At 0% D_2_O, the scattering density from the aqueous layers decreased sufficiently to reveal four distinct peaks (in the membrane pair, from 0 to *d*) corresponding to the lipid polar groups, which are relatively water-poor and hydrogen-poor and rich in more strongly scattering phosphorus, carbon and oxygen. Across all samples, and especially in rat PNS myelin, a shoulder was observed in the extracellular leaflet of the bilayer, proximal to the lipid polar group region. This asymmetry in neutron scattering density within the bilayer is consistent with the postulated enrichment of cholesterol in the extracellular leaflet. The steroid nucleus of cholesterol has a higher neutron scattering density (0.07 × 10^11^ cm^−2^) than stiff-chain hydrocarbon (−0.01 × 10^11^ cm^−2^) (Kirschner, 1974 ▶), which could account for its detection here.

The dimensions of the bilayers and intermembrane spaces were consistent with the measurements from previous XRD experiments. For both CNS and PNS samples, the size of the bilayer (determined by the distance from headgroup peak to headgroup peak) ranged from 40 to 46 Å, which is lower than the XRD measurements (where it is typically 46–47 Å), probably owing to the mixed contributions of the hydrogen-poor phosphates, glycerol backbones and fatty-acid ester linkages in the headgroup region (Franks & Lieb, 1979 ▶). The cytoplasmic compartments in all samples had similar widths, ranging from 32 to 37 Å, with no consistent differences between the PNS and CNS. However, the extracellular compartment varied significantly between the CNS and PNS, with the values of 32–40 Å for the CNS being similar to the cytoplasmic compartment width, whereas expanded values of 47–55 Å were measured in the PNS. The observed differences between rat and mouse myelin structures most likely result from differences in data quality rather than interspecies differences. For example, the relatively high scattering density of the extracellular compartment in mouse spinal cord (Fig. 1 ▶ *d*) compared with that of other samples probably comes from poor separation of the broad first-order reflection from the low-angle scatter near the beamstop, which may be improved in the future by using a narrower beam or thinner samples.

### 3.4. Hydrogen–deuterium exchange kinetics in myelin   

**Figure 6:**
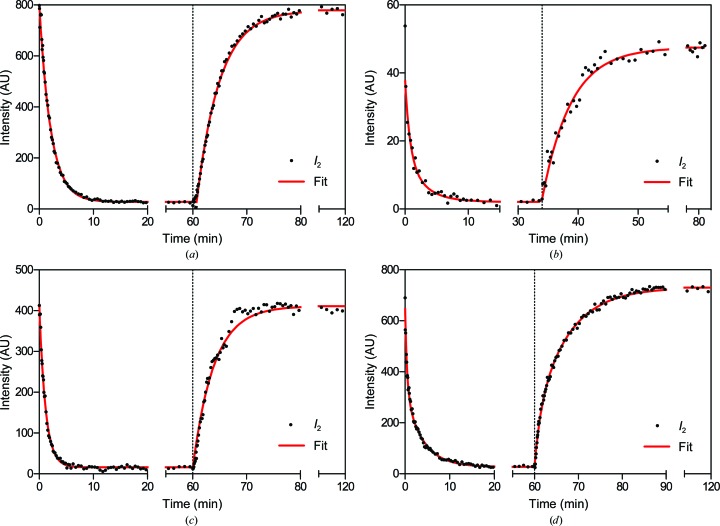
H_2_O–D_2_O exchange kinetics in (*a*) rat sciatic nerves, (*b*) rat optic nerves, (*c*) mouse sciatic nerves and (*d*) mouse spinal cords. Samples equilibrated against 100% D_2_O-saline were first perfused with 20% D_2_O-saline (*t* = 0), followed by a perfusion with 100% D_2_O-saline (arrow) once equilibrium was reached. The extent of exchange is indicated by the change in intensity of the second-order reflection over time. Long periods with no change are indicated by breaks in the *x* axis. Curves behind the data points represent double-exponential decay models fitted to the data.

To probe the insulative properties of myelin, as indicated by the accessibility of water and mobile ions to its multilamellar arrays, we measured water-exchange kinetics in rodent PNS and CNS myelin. Nervous-system tissue that had been equilibrated against buffered saline containing a known concentration of D_2_O was loaded into the perfusion chamber and continuously flushed *in situ* with saline containing a different concentration of D_2_O. Short, serial exposures were collected during perfusion, and the extent of H_2_O–D_2_O exchange was determined by measuring the change in intensity of the second-order Bragg reflection (which varies with the D_2_O content of myelin) over time (Fig. 6 ▶).

H_2_O–D_2_O exchange in rat sciatic nerves from high to low %D_2_O-saline (typically 100→20%) occurred rapidly, and the decrease in *F*_2_ followed a single exponential decay, with an average relaxation time (τ, or *t*_1/e_) of 3.17 ± 0.46 min (*n* = 3; Fig. 6 ▶ *a*, Table 2 ▶). This was significantly shorter than previously measured in rabbit sciatic nerves, which had relaxation times of between 6 and 12 min (Kirschner *et al.*, 1976 ▶). The longer times in the earlier study are likely to be accounted for by the 6°C temperature at which the nerves were maintained to preserve myelin integrity during long exposures. This temperature is only 2°C above the freezing point of pure D_2_O and results in a considerable increase in viscosity for both D_2_O and H_2_O (Hardy & Cottington, 1949 ▶), lowering the diffusion rates. Our measurements were always performed at ambient temperature, where the viscosities of D_2_O-saline and H_2_O-saline are substantially lower and diffusion is more rapid. Additionally, the relatively large size and lower surface area to volume ratio of bundled rabbit sciatic nerves could provide additional barriers to the free diffusion of water. Therefore, our new measurements are likely to be a more accurate indication of the exchange kinetics of water in myelin. The reverse exchange (20→100% D_2_O-saline) occurred more slowly than the forward exchange, with the relaxation times being 3.71 ± 0.39 min (*n* = 3; Fig. 6 ▶ *a*, Table 2 ▶). This difference is consistent with previous measurements and is most likely to be caused by the ∼25% higher viscosity of D_2_O compared with that of H_2_O (Hardy & Cottington, 1949 ▶).

H_2_O–D_2_O exchange in rat optic nerves mostly followed a double exponential decay, in contrast to the single exponential decay observed in sciatic nerves. The decrease in *F*_2_ for exchanges from high to low %D_2_O-saline proceeded with a primary relaxation time (τ_1_) of 3.97 ± 1.04 min (*n* = 5); for exchanges that showed a double exponential decay, an additional, very short relaxation time (*τ*_2_) of 0.44 ± 0.20 min (*n* = 4) was required to best fit the data (Fig. 6 ▶ *b*, Table 2 ▶). During the reverse exchange (from low to high %D_2_O-saline) the relaxation times were slightly longer, similar to the trend seen in the sciatic nerves. The reverse exchanges proceeded with a primary relaxation time of 4.75 ± 1.43 min (*n* = 2); in one experiment, however, a secondary relaxation time of 0.67 min (*n* = 1) was required to fit the data (Fig. 6 ▶ *b*, Table 2 ▶). The apparent inconsistencies in the exponential modelling of H_2_O–D_2_O exchange in rat optic nerves can be explained by the relatively weak diffraction (Fig. 3 ▶) combined with the fact that many of the exchanges measured were performed between similar and often low levels of D_2_O-saline (*e.g.* 60→43% and 43→20% D_2_O). Higher quality data from mouse spinal cord (below), however, validated our analysis here.

The water-exchange rates determined for mouse sciatic nerves were consistent with the data from rat sciatic nerves: exchange from 100 to 20% D_2_O-saline followed a single exponential decay with a relaxation time of 1.72 min (*n* = 1), while the reverse exchange (20→100% D_2_O-saline) showed a relaxation time of 2.47 min (*n* = 1; Fig. 6 ▶ *c*, Table 2 ▶). Exchange experiments were not performed on mouse optic nerves because of their weak diffracting power (Fig. 3 ▶). Instead, mouse spinal cords were examined. During exchange from 100 to 20% D_2_O-saline, these samples displayed a decrease in *F*_2_ that consistently showed a double exponential decay, with a primary relaxation time of 5.84 ± 1.15 min and a secondary relaxation time of 0.67 ± 0.27 min (*n* = 6; Fig. 6 ▶ *d*, Table 2 ▶). The reverse exchange (20→100% D_2_O-saline) was significantly slower, showing primary and secondary relaxation times of 7.48 ± 1.11 min (*p* < 0.05) and 0.71 ± 0.03 min (*n* = 6), respectively. Taken as a whole, the relaxation times were on average about 25% longer for exchanges from low to high %D_2_O than from high to low %D_2_O (*n* = 6; paired *t*-test; *p* < 0.04).

### 3.5. The effect of heavy water on myelin structure   

**Figure 7:**
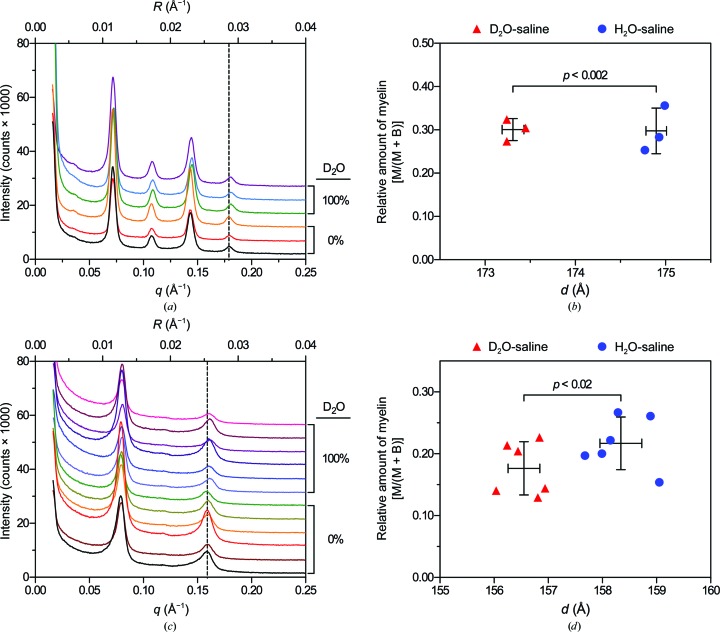
Subtle effect of heavy water on the myelin period. (*a*, *c*) X-ray diffraction patterns collected from mouse (*a*) sciatic nerves and (*c*) spinal cord segments equilibrated against either H_2_O-saline or D_2_O-saline. Scattering intensity is plotted against both the scattering vector *q* (Å^−1^) and the reciprocal coordinate *R* (Å^−1^). For clarity, the spectra have been offset along the *y* axis. For comparison, the dashed lines represent the average positions of the fifth-order and fourth-order reflection from sciatic nerves and spinal cords, respectively, in H_2_O-saline. (*b*, *d*) Scatter plots of relative amount of myelin [M/(M + B)] *versus* myelin period *d* for mouse sciatic nerves (*b*) and spinal cords (*d*) equilibrated against either H_2_O-saline or D_2_O-saline. Error bars represent ±1 standard deviation centred on the average for each group. Statistical significance was calculated using a paired *t*-test on parameters measured from single sciatic nerves or averaged parameters from two segments of spinal cord isolated from each mouse (*n* = 3) split between each of the two treatments.

It has previously been reported that frog (*Rana pipiens*) PNS myelin in H_2_O-saline produces identical XRD patterns to nerves soaked in D_2_O-saline (Akers & Parsons, 1970 ▶). However, throughout our ND experiments, we noticed that the samples exhibited a slight, but constant, tendency towards a lower period as the concentration of D_2_O increased. This difference was typically no greater than 4 Å between nerves in 100% H_2_O-saline *versus* nerves in 100% D_2_O-saline (data not shown). To test whether deuterium was causing unexpected structural changes, we soaked nerves in H_2_O-saline and D_2_O-saline and analyzed them using XRD, which does not distinguish between deuterium and hydrogen. We found that mouse sciatic nerves in D_2_O-saline displayed a 1.6 Å lower period in D_2_O-saline, 173.3 ± 0.1 Å, than in H_2_O-saline, 174.9 ± 0.1 Å (*n* = 3; paired *t*-test; *p* < 0.002; Figs. 7 ▶ *a* and 7 ▶ *c*). Similarly, the myelin period in mouse spinal cord was 1.8 Å lower in D_2_O-saline, 156.6 ± 0.3 Å, than in H_2_O-saline, 158.3 ± 0.4 Å (*n* = 3, paired *t*-test, *p* < 0.02; Figs. 7 ▶ *b* and 7 ▶ *d*). Close examination and direct measurement of the relevant diffraction pattern in question (Akers & Parsons, 1970 ▶) revealed an apparent 2 Å expansion of frog PNS myelin in D_2_O-saline that was unreported by the investigators. It is unclear whether the authors controlled for the difference in p*K*_a_ between H_2_O and D_2_O. If not, the increased alkalinity of D_2_O-saline (by 0.4 units) could account for the observed increase in period (Akers & Parsons, 1970 ▶); similarly, a slight increase in ionic strength owing to the additional acid used to equalize the pH/pD of H_2_O and D_2_O-containing buffers may account for the compaction observed in the present study (Inouye & Kirschner, 1988 ▶; Worthington & Blaurock, 1969 ▶; Finean & Millington, 1957 ▶; Worthington, 1979 ▶; Robertson, 1958 ▶). Although these changes only represent a ∼1% reduction in period, these results emphasize the dynamic nature of myelin and demonstrate the ability of diffraction-based methods to detect subtle changes in structure.

## 4. Discussion   

Early EM studies of myelin revealed a significant amount of structural complexity (Schnapp & Mugnaini, 1975 ▶), and with the development of advanced imaging techniques and molecular-biology in the last few decades, researchers have been able to identify and localize the molecular constituents of myelin (Trapp & Kidd, 2004 ▶; Arroyo & Scherer, 2000 ▶; Scherer & Arroyo, 2002 ▶). Examining the molecular organization, structural dynamics and structure–function relationships in myelin, however, remains challenging because of the nanometre scale of the structural features of myelin. Of the methods that have sufficient resolving power, EM is the most prevalent; unfortunately, owing to processing artifacts, electron micrographs are rarely faithful representations of the native state of the tissue. For example, EM may give a false impression that myelin is a static structure by virtue of the uniformly static images that it produces. Additionally, the chemical and physical treatments that are routinely, and necessarily, applied to myelin prior to EM analysis can obscure or alter the structural features of myelin (Moretz *et al.*, 1969*a* ▶,*b* ▶; Kirschner & Hollingshead, 1980 ▶; Avila *et al.*, 2005 ▶). A compelling illustration of this is the once-held belief that myelin was entirely devoid of transmembrane proteins, as shown by the smooth membrane surfaces observed in freeze–fracture EM (Branton, 1967 ▶); this was later discovered to be the artifactual result of insufficient equilibration of cryoprotectants in the multilamellar structure (Kirschner *et al.*, 1979 ▶; Hollingshead *et al.*, 1981 ▶). Other misconceptions, however, persist. Among them is the pervasive usage of the term ‘major dense line’ to refer to the cytoplasmic apposition of myelin. Although commonplace, this term derives from the highly compacted and dense appearance of this space in electron micrographs, which is caused by osmication and dehydration during conventional tissue processing (Kirschner & Hollingshead, 1980 ▶). In untreated tissue, the cytoplasmic and extracellular spaces of CNS myelin are similar in size and electron density. These, and other treatments involved in tissue processing, are also responsible for the ∼20–30% reduction in the myelin period typically measured from electron micrographs compared with that determined by XRD experiments on fresh tissue. Issues pertaining to accurate determinations and depictions of myelin structure are important, as the size and accessibility of the cytoplasmic space are implicated in myelin biogenesis and the maintenance of myelin organization (Aggarwal *et al.*, 2011 ▶, 2013 ▶; Snaidero *et al.*, 2014 ▶). Recent advances in high-pressure freezing and freeze-substitution have improved tissue preservation with respect to antigen preservation (Kirschning *et al.*, 1998 ▶); however, even these methods eventually rely on chemical fixation and fail to maintain important features of the myelin structure (*i.e.* the myelin period and intermembrane packing distances; Möbius *et al.*, 2008 ▶, 2010 ▶).

To understand myelin biogenesis, architecture and dynamics, an accurate view of the myelin structure is essential for interpreting recent developments and informing future work. For these reasons, diffraction-based methods are often a necessary complement to the microscopic analysis of myelin; in diffraction experiments, samples can be analyzed fresh and immediately after dissection, completely avoiding the artifacts introduced by tissue processing. Diffraction methods largely depend on the presence of periodic structure within the sample; therefore, the repetitive nature of myelin membrane packing and other nervous-system assemblies (*e.g.* the radial component, paranodal loops, paranodal junctions, neurofilaments, microtubules and collagen) make the nervous system a highly amenable target for diffraction. The challenge lies in tailoring the experimental setup (*i.e.* radiation type; beam characteristics; detector efficiency, resolution and placement; and sample preparation and orientation) to highlight the features of interest. For example, the recent use of a 1 µm diameter X-ray microbeam to study membrane packing in internodal, juxtaparanodal and paranodal myelin in single myelinated nerves as well as the in-plane aggregation of P0 glycoprotein *in situ* aptly demonstrates the power of diffraction-based methods (Inouye *et al.*, 2014 ▶).

Here, we explored the use of ND as a potential method for myelin structural analysis. Compared with the more common XRD, which measures periodic fluctuations in electron density, ND measures variations in nuclear structure (neutron scattering density) within the sample and is especially sensitive to the presence of deuterium, a heavy isotope of hydrogen (Bacon & Lonsdale, 1953 ▶). Thus, if a defined population of H atoms within a biological sample can be replaced (either partially or completely) with deuterium, the position of this substitution can be determined with high accuracy (Büldt *et al.*, 1978 ▶, 1979 ▶; Zaccai *et al.*, 1975 ▶, 1979 ▶; Worcester & Franks, 1976 ▶). In our experiments, we performed a bulk exchange in rodent nervous tissue with buffers containing mixtures of light water (H_2_O) and heavy water (D_2_O) and analyzed the samples using ND. Through this simple replacement, we were able to localize readily exchangeable H atoms (from both bulk water and ionizable chemical groups), determine myelin structural parameters and measure H–D exchange kinetics in CNS and PNS myelin.

Our ND experiments on sciatic nerve myelin revealed structural details that were not seen in previous experiments from ∼45 years ago (Parsons & Akers, 1969 ▶). Originally, only a single second-order reflection was observed from both rabbit and human peripheral nerves in 100% D_2_O-saline, suggesting a simple, cosinusoidal distribution of neutron scattering density in myelin that corresponded to the alternating extracellular and cytoplasmic layers of exchangeable water (Parsons & Akers, 1969 ▶); however, this model lacked any fine structural detail. Subsequent work using rabbit sciatic nerve in 100% D_2_O-saline revealed additional Bragg orders (1–4, 6 and 7), which contributed to a more detailed neutron scattering density profile (Kirschner *et al.*, 1976 ▶). In our current ND experiments, which used the considerably smaller sciatic nerves from rat and mouse, we typically observed Bragg orders 1–7, and also recorded orders 1–6 and 8 from rat sciatic nerves in 100% H_2_O-saline, despite the strong incoherent neutron scattering signal from H_2_O. A previous ND study using human sciatic nerves in H_2_O was unable to detect any coherent neutron scatter from the sample (Parsons & Akers, 1969 ▶). Additionally, we analyzed CNS myelin (rat optic nerves and mouse spinal cord) and recorded Bragg orders 1, 2, 4 and 6 in D_2_O and orders 1 and 4 in H_2_O; previously, only a single second-order reflection had been observed from samples in D_2_O (Worcester, 1976 ▶). Notably, all analyses were performed on samples from single animals, minimizing the influence of inter-animal variation for each measurement. Furthermore, the exposure times in our static experiments were 1–5 h, significantly shorter than the 8 h to 4 d exposures previously used (Kirschner *et al.*, 1976 ▶).

Neutron scattering density profiles from rodent CNS and PNS myelin demonstrated the familiar double-membrane bilayer organization of the myelin sheath in the internode. At low %D_2_O, the membrane-bilayer hydrocarbon core, polar headgroup peaks and aqueous compartments were clearly delineated. For all samples, the distance across the bilayer was 40–46 Å, while the cytoplasmic compartment was measured to be 32–37 Å; the width of the extracellular space varied between CNS (32–40 Å) and PNS (47–55 Å) myelin. These dimensions are consistent with numerous XRD experiments on myelin (reviewed in Kirschner & Blaurock, 1992 ▶). At high %D_2_O, the similarity in water content of the two aqueous compartments of myelin was apparent. This is in stark contrast to the condensed nature of the major dense line as observed in EM. It is commonly written that the major dense line is formed by the fusion of cytoplasmic leaflets of the oligo­dendrocyte/Schwann-cell membrane and is therefore devoid of cytoplasm; however, our measurements clearly demonstrate that the cytoplasmic apposition is similar to the extracellular apposition in terms of width (CNS) and water content (CNS and PNS), and could harbour small metabolites and other molecules in an aqueous environment.

Our analysis of the ND data also revealed more subtle variations in membrane structure. In particular, we measured an apparent asymmetry in the bilayer, as shown by an increased neutron scattering density in the extracellular leaflet. The higher scattering density of the extracellular leaflet may represent an enrichment of cholesterol on this side of the membrane, as the steroid nucleus of cholesterol has a higher neutron scattering density than stiff-chain hydrocarbon: 0.07 × 10^11^ *versus* −0.01 × 10^11^ cm^−2^, respectively (Kirschner, 1974 ▶). The asymmetric distribution of cholesterol in myelin was suggested previously by high-resolution X-ray diffraction studies (Caspar & Kirschner, 1971 ▶).

In addition to static measurements performed on freshly dissected samples in sealed capillaries, we also used ND to measure H_2_O–D_2_O exchange kinetics in samples that were continuously perfused with fluid. These experiments required the collection of a series of short, consecutive exposures. H_2_O–D_2_O exchange was monitored using exposure times less than one-tenth as long as previously used for samples that were as much as tenfold greater in size, demonstrating the improved capabilities of current neutron technology. Analysis of H_2_O–D_2_O exchange revealed consistent differences between CNS and PNS myelin. On average, exchange in PNS tissue was almost twice as rapid as that in CNS tissue (τ_1_; *n* = 4; *p* < 0.03). Furthermore, exchange generally proceeded with a single exponential decay in PNS myelin, whereas in CNS myelin double-exponential fits were required to model the exchange data. This difference between CNS and PNS myelin indicates the existence of two somewhat distinct populations of exchangeable H atoms within myelin: the rapidly exchanging bulk water and the more slowly exchanging interfacial water and labile H atoms bound to macromolecules, or populations of water/hydrogen in either the cytoplasmic or extracellular compartments. Identification of these populations may reveal important insights into the water and solute permeability of internodal myelin.

The current study demonstrates the renewed potential for ND studies on myelin and other natural biological membranes. Although the simple *ex vivo* exchange of water performed here provides valuable insight into the distribution and accessibility of water within myelin, alternative routes of administration and/or the use of other deuterated molecules will facilitate the tackling of additional questions. For example, simple *ex vivo* treatment of myelin could address the interactions of biologically relevant deuterated small molecules with myelin (*e.g.* anesthetics, organic solvents or fixatives such as glutaraldehyde). More elaborate studies could involve the *in vivo* incorporation of deuterium into the membranes during myelination. Because mice can tolerate >30% D_2_O in their drinking water indefinitely, one can expect >15% replacement of hydrogen by deuterium in newly synthesized lipids and proteins (Katz *et al.*, 1962 ▶; Ando *et al.*, 2003 ▶) through a simple feeding protocol. This broad labelling technique should impart sufficient contrast to highlight the hydrogen-rich hydrocarbon core of the bilayer and to allow its dimensions and internal distribution of hydrogen to be examined. A similar approach could be taken to analyze the distribution of single lipid species within the membrane. For example, the proposed asymmetric distribution of cholesterol could be addressed through the administration of deuterated cholesterol to pregnant dams and then postnatally, enabling the labelled cholesterol to become incorporated into tissues during the early development and maturation of the pups (Woollett, 1996 ▶; Scott *et al.*, 1980 ▶; Wechsler *et al.*, 2003 ▶). Similarly, the use of deuterated lipid precursors (*e.g.* ketone bodies, mevalonate and fatty acids) or targeted delivery techniques may allow the appropriation of endogenous synthesis pathways and confinement of the label to a tissue of interest (Chevallier & Gautheron, 1969 ▶; Edmond, 1974 ▶; Sun & Horrocks, 1973 ▶; Gozlan-Devillierre *et al.*, 1978 ▶).

Beyond elucidating the structure of myelin, neutron diffraction holds much promise in analyzing the function of myelin. The monitoring over time of a simple exchange of deuterated and protonated material provides a direct and focused measure of internodal myelin function and integrity, in contrast to the bulk electrophysiological properties of myelinated tissue that is often used to interrogate myelin function. The uncoupling of myelin function and nerve conduction allows myelin to be studied in relative isolation, which could be exploited to measure the permeability of not only healthy myelin but also myelin from transgenic animals and animal models of human myelinopathies. For example, mice lacking components of the axo–glial junctions or interlamellar tight junctions and animal models of multiple sclerosis, such as the experimental autoimmune encephalomyelitis (EAE) mouse, would be of particular interest. A forthcoming manuscript will focus on our use of ND and XRD to characterize myelin from mice lacking the CNS interlamellar tight junction protein claudin-11 (Gow *et al.*, 1999 ▶; Devaux & Gow, 2008 ▶).

## Figures and Tables

**Table 1:** Structure factors for rat and mouse PNS and CNS myelin *versus* %D_2_O

		Average *F_h_* with assigned phase and uncertainty†					
Sample	Bragg order *h*	100% D_2_O	80% D_2_O	60% D_2_O	43% D_2_O	20% D_2_O	0%D_2_O
Rat sciatic nerve	1	0.138 (0.002)	0.087 (0.002)	0.068 (0.003)	0.049 (0.004)		0.009 (0.001)
	2	1.000 (0.003)	0.796 (0.003)	0.593 (0.005)	0.419 (0.005)	0.185 (0.003)	0.017 (0.001)
	3	0.169 (0.004)	0.129 (0.004)	0.096 (0.006)	0.069 (0.006)	0.039 (0.004)	0.017 (0.002)
	4	0.081 (0.004)	0.100 (0.005)	0.094 (0.007)	0.095 (0.007)	0.090 (0.004)	0.095 (0.002)
	5	0.054 (0.006)					0.051 (0.002)
	6	0.123 (0.005)	0.106 (0.006)	0.088 (0.008)	0.049 (0.009)	0.049 (0.005)	0.036 (0.003)
	7	0.081 (0.006)	0.058 (0.006)	0.062 (0.009)	0.048 (0.010)	0.041 (0.005)	
	8						0.0339 (0.003)
Rat optic nerve	1	0.071 (0.003)	0.045 (0.003)	0.048 (0.003)	0.050 (0.002)	0.045 (0.004)	0.020 (0.001)
	2	1.000 (0.004)	0.805 (0.005)	0.609 (0.005)	0.443 (0.002)	0.219 (0.006)	
	4	0.062 (0.006)	0.032 (0.006)		0.060 (0.003)	0.081 (0.008)	0.076 (0.002)
	6	0.111 (0.007)	0.113 (0.008)	0.108 (0.008)	0.050 (0.004)		
Mouse sciatic nerve	1	0.151 (0.004)	0.126 (0.005)	0.079 (0.004)	0.072 (0.005)		
	2	1.000 (0.006)	0.794 (0.007)	0.587 (0.006)	0.412 (0.007)	0.175 (0.005)	
	3	0.169 (0.007)	0.147 (0.008)	0.127 (0.008)	0.098 (0.009)		
	4	0.075 (0.008)	0.084 (0.009)	0.105 (0.009)	0.107 (0.010)	0.096 (0.007)	
	5	0.057 (0.009)	0.069 (0.010)		0.073 (0.012)		
	6	0.122 (0.010)	0.091 (0.011)	0.073 (0.011)			
	7	0.061 (0.010)	0.065 (0.012)	0.053 (0.012)			
Mouse spinal cord	1	0.236 (0.002)			0.083 (0.001)		
	2	1.000 (0.003)			0.438 (0.002)		
	4	0.033 (0.006)			0.048 (0.004)		
	6	0.092 (0.007)					

† Uncertainty in *F_h_* is denoted by counting statistics (values in parentheses).

**Table 2:** Relaxation times for H_2_OD_2_O exchange in rat and mouse PNS and CNS myelin

	Relaxation time (min)	High to low %D_2_O	Low to high %D_2_O
Rat sciatic nerve	_1_	3.17 0.46 (*n* = 3)	3.71 0.39 (*n* = 3)
Rat optic nerve	_1_	3.97 1.04 (*n* = 5)	4.75 1.43 (*n* = 2)
	_2_	0.44 0.20 (*n* = 4)	0.67 (*n* = 1)
Mouse sciatic nerve	_1_	1.72 (*n* = 1)	2.47 (*n* = 1)
Mouse spinal cord	_1_	5.84 1.15 (*n* = 6)	7.48 1.11 (*n* = 6)
	_2_	0.67 0.27 (*n* = 6)	0.71 0.03 (*n* = 6)
